# Behavior of fast and slow myosin in diabetic rats submitted to resistance power exercise

**DOI:** 10.1186/1758-5996-7-S1-A9

**Published:** 2015-11-11

**Authors:** Cybelle da Silva Nery, Marcos Paulo Galdino Coutinho, Deniele Bezerra Lós, Kamilla Dinah Santos de Lira, Filipe Barbosa Cunha de Miranda, Ana Carolina Salvador de Lucena, Ana Camila Nobre de Lacerda Brito, Silvia Luana Ramos Marques, Márcio Almeida Bezerra, Sílvia Regina Arruda de Moraes

**Affiliations:** 1 Universidade Federal De Pernambuco, Brazil

## Background

Myopathy is a recurrent change in diabetic state, characterized by muscle atrophy, weakness and decreased physical capacity (Andersen; Schmitz; Nielsen, 2005). Atrophy in diabetic state is the result of decreased insulin (Dall'ago et al., 2002) which results in the inhibition of myosin synthesis (Vandenburgh et al., 1991), protein responsible for the contractile properties of the muscle which is shaped according to stimulate the muscle is exposed (Iorga; Adamek; Geeves, 2007). In turn, exercise has the ability to reverse the phenotypic changes in mitochondrial muscle proteins caused by insulin deficiency states (Midaoui; Tancrede; Nadeau, 1996).

## Objective

To evaluate the distribution of fast and slow myosin in the lateral gastrocnemius muscle of rats submitted to a resistance training.

## Materials and methods

22 Wistar rats were used, 60 years old, divided into four groups: Sedentary Control Group-SCG (n=3), Sedentary Diabetic Group-SDG (n=8), Trained Control Group-TCG (n=3), Trained Diabetic Group-TDG (n=8). Induction of diabetes was made by streptozotocin. The protocol of resistance jump exercise took nine weeks (ROGATTO; LUCIANO, 2001). Blood glucose and body weight of the animals from the beginning to the end of the experiment were evaluated. With the end of the exercise protocol, animals' right side gastrocnemius was collected and sent to immunohistochemistry to quantify the fast and slow myosin.

## Results

Animals of all diabetic groups presented lower final body weight (p <0.05) and blood glucose values greater than 200 mg/dL from induction to the end of the experiment compared to controls. It was observed that the lateral gastrocnemius muscle weights were shown to be reduced in the SDG when compared to SCG (p <0.05), increased in the TCG when compared to SCG (p <0.05) and there is no difference between the comparison of the animals of SDG and TDG (p> 0.05). The analysis of fast and slow myosin by immunohistochemistry, it had not demonstrated any difference between groups (p> 0.05).

## Conclusion

Diabetic myopathy did not alter the normal distribution of fast and slow myosin in the lateral gastrocnemius muscle, and resistance training had no effect on the distribution of this protein.

